# Comparison of calcium-based technologies to remineralise enamel subsurface lesions using microradiography and microhardness

**DOI:** 10.1038/s41598-022-13905-8

**Published:** 2022-06-14

**Authors:** James R. Fernando, Glenn D. Walker, Thomas Kwan-Soo Park, Peiyan Shen, Yi Yuan, Coralie Reynolds, Eric C. Reynolds

**Affiliations:** Centre for Oral Health Research, Melbourne Dental School, Bio21 Institute, The University of Melbourne, Melbourne, Australia

**Keywords:** Diseases, Health care, Materials science

## Abstract

Assessment of enamel subsurface lesion remineralisation is essential for the evaluation of novel remineralisation technologies. The gold standard to assess subsurface mineral gain of enamel lesions is transverse microradiography (TMR). However, some studies have utilised surface microhardness (SMH) to evaluate efficacy of remineralisation agents. The aim of this study was to assess remineralisation of enamel subsurface lesions using TMR and SMH after in vitro treatment with calcium-containing technologies, and to test correlation between the TMR and SMH measurements. The parameters obtained from the TMR and SMH analyses of enamel subsurface remineralisation were not significantly correlated. Furthermore, the enamel subsurface remineralisation as measured by TMR was significantly correlated with the water-soluble calcium concentration of the remineralisation products. Scanning electron microscopy revealed surface precipitates formed by specific remineralisation treatments obfuscated accurate assessment of remineralisation by SMH. It was concluded that TMR is a more appropriate method for analysis of enamel subsurface remineralisation, and that SMH values of remineralised enamel should be interpreted with caution. Using TMR the level of remineralisation (%R) by the different technologies was CPP-ACP/F (31.3 ± 1.4%); CPP-ACP (24.2 ± 1.4%); CaSO_4_/K_2_HPO_4_/F (21.3 ± 1.4%); f-TCP/F (20.9 ± 1.0%); Nano-HA/F (16.3 ± 0.3%); Nano-HA (15.3 ± 0.6%) and F alone control (15.4 ± 1.3%).

## Introduction

Promoting remineralisation of mineral-deficient tooth structure is fundamental to caries management in modern dentistry^1,2^. Remineralisation occurs by the diffusion of soluble, bioavailable calcium and phosphate ions, externally sourced, through the nanoporosities of the enamel surface layer into the subsurface lesion and by the deposition of those ions into apatite crystal voids within the lesion^3^. Despite being an important source of ions for remineralisation, saliva alone has a relatively low potential to remineralise carious tooth structure^4,5^. Anticariogenic fluorides are potent drivers of remineralisation, having contributed to the worldwide reduction in caries prevalence over the twentieth century since their introduction^4,6^. However, fluoride-promoted remineralisation occurs primarily in the superficial region of carious lesions and is limited by the bioavailability of calcium and phosphate ions present in saliva and plaque^7,8^. Hence, in recent decades there have been efforts to develop calcium- and phosphate-containing remineralisation technologies, such as casein phosphopeptide-stabilised amorphous calcium phosphate, nano-hydroxyapatite, calcium phosphosilicates and functional tri-calcium phosphate^8,9^.

Accurate assessment of remineralisation ex vivo is important for developing remineralisation strategies, and numerous methods have been suggested^10,11^. Widely accepted as the ‘gold standard’ method, transverse microradiography (TMR) utilises x-rays to quantify volumetric mineral gain in enamel expressed as percent remineralisation (%R)^11–13^. While TMR is deemed a reliable and reproducible method to quantify subsurface mineral distribution, the sample is destroyed in the process and the analysis requires access to specific equipment of appropriate sensitivity^14^. As an adjunct, microhardness assessment of surface enamel (SMH) has been used to evaluate mechanical properties following remineralisation^15^. This technique preserves the sample and allows for longitudinal assessment, measuring mechanical changes to superficial tooth structure^16^. Accordingly, previous authors have reported percent surface microhardness recovery (%SMHR) of treated erosive lesions^16–21^, and treated subsurface lesions when combined with mineral density measurements from TMR^15,22–24^. However, some studies have also used %SMHR to predict and differentiate subsurface remineralisation^25–29^.

The primary aim of this study was to compare the use of TMR and %SMHR for assessment of remineralisation of enamel subsurface lesions, and the secondary aim was to compare the effect of a range of calcium-containing remineralisation products (Table 1) in vitro. Standardised laboratory-produced enamel subsurface lesions in human teeth were treated with remineralisation products diluted in artificial saliva. Using measurements obtained from SMH and TMR, the differences between remineralisation treatments were tested using a one-way analysis of variance, and correlation between %SMHR and %R measurements was also assessed. The null hypotheses were that %R and %SMHR would be significantly correlated and that no significant differences in %R would be found between remineralisation treatments. Scanning electron microscopy (SEM) imaging was used to characterise the surface of treated enamel, and ion chromatography was also used to determine water-soluble calcium levels in the remineralisation solutions.

**Table 1:** Remineralisation treatments: active ingredients, fluoride concentration and dilution factor in artificial saliva (AS).

Group	Remineralisation solution	Manufacturer	Active ingredients^a^	Total fluoride (ppm)
A	Artificial Saliva (AS)	–	–	–
B	AS + Colgate Total	Colgate	0.22% w/w NaF	1000
C	AS + Tooth Mousse	GC	CPP-ACP	–
D	AS + Tooth Mousse Plus	GC	CPP-ACP	900
			0.2% w/w NaF	
E	AS + Age Defying	Arm & Hammer	0.24% w/w NaF	1100
			Calcium Sulfate	
			Dipotassium phosphate	
F	AS + Apagard m-Plus	Sangi	Nano HA	–
G	AS + Remin Pro	Voco	Nano HA	1450
			0.32% w/w NaF	
H	AS + Clinpro Tooth Crème	3 M ESPE	f-TCP	950
			0.21% w/w NaF	

*CPP-ACP* casein phosphopeptide-amorphous calcium phosphate, *Nano-HA* nanohydroxyapatite, *f-TCP* functional tricalcium phosphate.

## Results

The analysis of the water-soluble calcium concentrations of the remineralisation solutions (Table 2) indicated that the stabilised calcium phosphate products (Tooth Mousse and Tooth Mousse Plus) contained the highest water-soluble calcium concentration, over 10 times that of any other remineralisation solution at a similar dilution. The lowest water-soluble calcium of the calcium-containing products was observed with the Remin Pro solution, which contained nano-hydroxyapatite and sodium fluoride.

**Table 2:** Water-soluble calcium concentrations in diluted treatment solutions measured with ion chromatography.

Remineralisation solution	Water-soluble Ca (mM)
AS	0.50
AS + Colgate Total	0.61
AS + Tooth Mousse	24.75
AS + Tooth Mousse Plus	25.05
AS + Age Defying	2.39
AS + Apagard M-Plus	1.61
AS + Remin Pro	0.70
AS + Clinpro Tooth Crème	1.05

**Figure 1:**
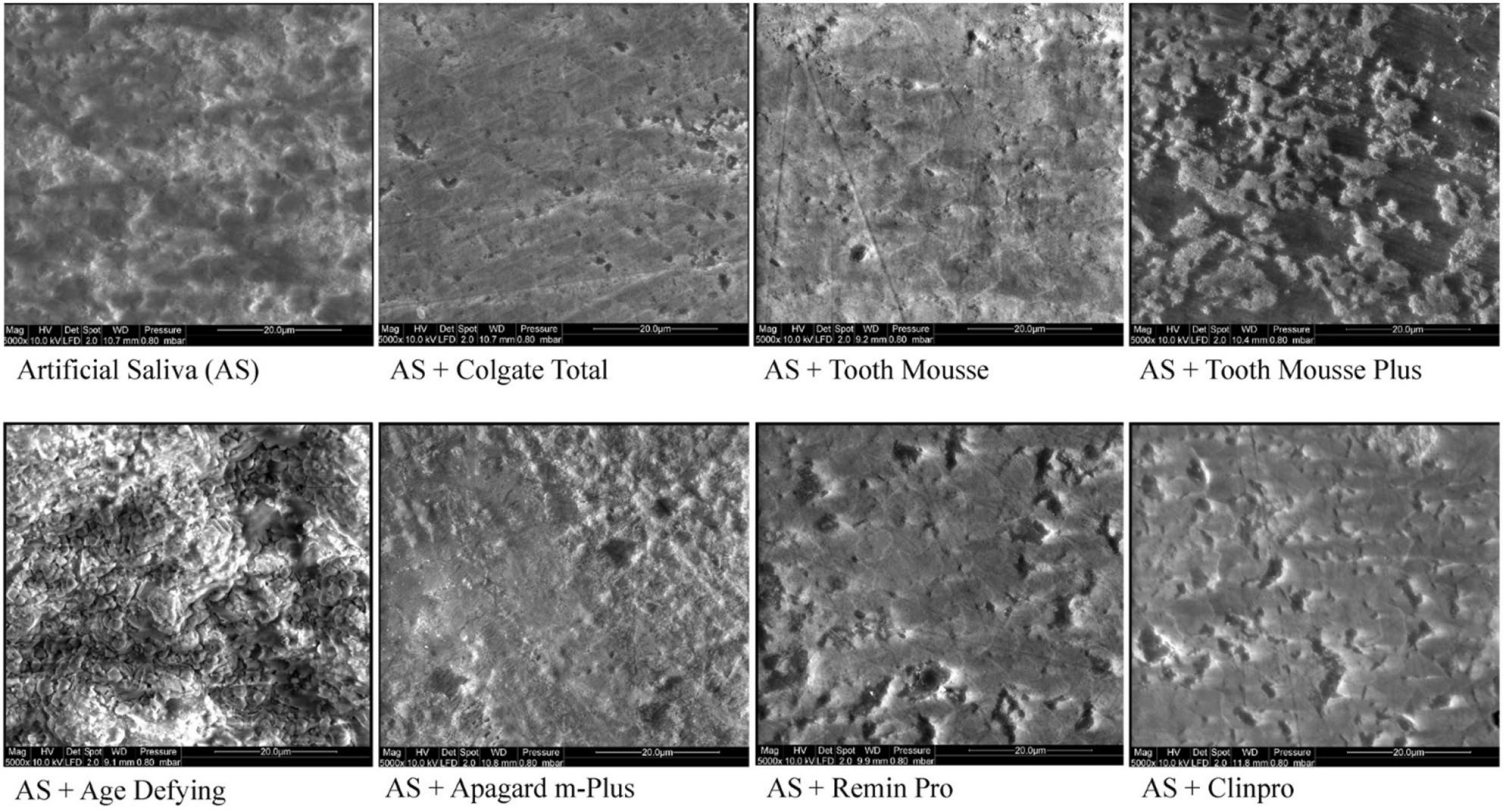
Representative enamel surface SEM images of treated subsurface lesions (× 5000 magnification).

No significant differences between groups before treatment were observed for mean VHN_s_ and VHN_d_. Significant differences were noted after treatment for mean VHN_r_ and %SMHR (see Table 3). As expected, the lowest mean %SMHR was observed in the control (AS) group. The highest mean %SMHR was observed in the group treated with the Age Defying remineralisation solution, which contained calcium sulfate and dipotassium phosphate. Relatively high %SMHR was also observed in the nano-hydroxyapatite-containing remineralisation solution with Apagard m-Plus. Figure 1 shows representative SEM images of the treated enamel surfaces. Many of the enamel lesions developed surface precipitates following treatment which made visualisation of microhardness indentations difficult. The most significant precipitates were observed on lesions exposed to the Age Defying remineralisation solution.

**Table 3:** Surface microhardness recovery of subsurface enamel lesions by treatment solutions.

Remineralisation solution	VHN_s_*	VHN_d_^α^	VHN_r_^β^	%SMHR^γ^
AS	319.34 ± 9.75	61.41 ± 8.92	71.49 ± 8.40^a^	3.92 ± 1.44^a^
AS + Colgate Total	325.64 ± 12.03	59.68 ± 8.39	82.42 ± 7.86^ab^	8.61 ± 1.90^b^
AS + Tooth Mousse	328.37 ± 5.14	57.85 ± 9.60	81.08 ± 11.37^ab^	8.87 ± 2.27^b^
AS + Tooth Mousse Plus	326.26 ± 10.11	59.68 ± 11.44	79.54 ± 10.55^ab^	7.48 ± 1.59^b^
AS + Age Defying	325.59 ± 25.43	64.78 ± 8.26	132.69 ± 33.3^c^	27.47 ± 12.13^c^
AS + Apagard m-Plus	315.70 ± 8.98	60.69 ± 9.95	97.19 ± 12.89^bc^	14.38 ± 2.21^c^
AS + Remin Pro	318.49 ± 9.27	67.82 ± 7.41	78.86 ± 7.40^ab^	4.43 ± 2.06^a^
AS + Clinpro Tooth Crème	316.83 ± 21.70	63.24 ± 7.03	78.61 ± 7.47^ab^	6.12 ± 1.30^ab^

*VHN_s_: Kruskal–Wallis test—no difference across treatments (p > 0.05).

^α^VHN_d_: ANOVA—no difference across treatments (p > 0.05).

^β^VHN_r_: ANOVA—significant difference across treatments (p < 0.0001).

^γ^%SMHR: ANCOVA—main effects model for treatment and VHN_s_–VHN_d_ as covariate.

Significant differences across treatments (p < 0.0001) with significant effect of VHN_s_–VHN_d_ as covariate (p = 0.042).

^abc^Same superscripts in column denote means that are not significantly different (p > 0.05). All differences between treatments were measured using pairwise comparisons with a Sidak adjustment.

**Figure 2:**
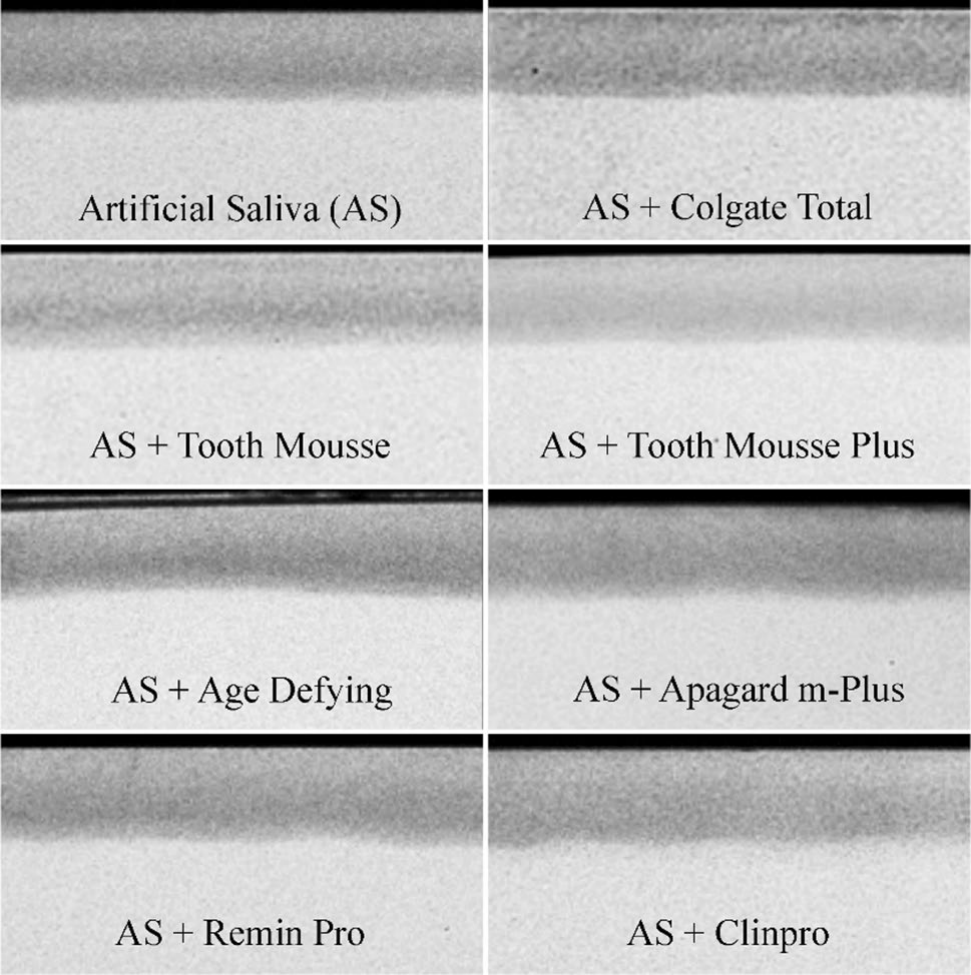
Representative transverse microradiographs of treated enamel subsurface lesions.

Table 4 displays results from the TMR analysis. No significant differences were noted between groups before treatment for LD_d_, LD_d_–LD_r_, and ZD_d_. For %R after treatment, the Colgate Total, Apagard m-Plus and Remin Pro remineralisation solutions were not significantly different, and the Age Defying and Clinpro Tooth Crème remineralisation solutions were also not significantly different. %R was observed to be significantly different between all other remineralisation solutions (p < 0.0001). Hence, this null hypothesis was rejected. The highest and second highest %R were observed in lesions treated with remineralisation solutions containing stabilised calcium; Tooth Mousse Plus (31.33 ± 1.38%) and Tooth Mousse (24.21 ± 1.39%), respectively. The lowest %R was observed in lesions treated by AS only. Figure 2 displays representative microradiographs of treated subsurface lesions for each solution. Surface radiopacities were visible on the microradiographs of lesions treated by the Age Defying remineralisation solution.

**Table 4:** Remineralisation of enamel subsurface lesions by treatment solutions.

Remineralisation solution	LD_d_ (µm)*	LD_d_–LD_r_ (µm)^α^	ZD_d_ (vol% min µm)^β^	ZD_d_–ZD_r_ (vol% min µm)^γ^	%R^δ^
AS	89.65 ± 2.93	1.79 ± 2.22	1989.59 ± 276.35	226.23 ± 37.45	11.37 ± 1.26
AS + Colgate Total	88.98 ± 3.62	4.01 ± 2.52	2236.00 ± 284.69	344.02 ± 57.79^ab^	15.37 ± 1.28^ab^
AS + Tooth Mousse	91.69 ± 1.62	3.87 ± 1.45	2294.78 ± 164.23	554.56 ± 35.40	24.21 ± 1.39
AS + Tooth Mousse Plus	92.44 ± 1.18	4.04 ± 0.86	2469.07 ± 134.93	773.96 ± 58.06	31.33 ± 1.38
AS + Age Defying	89.65 ± 2.39	1.11 ± 2.52	2137.87 ± 342.92	451.07 ± 46.65^c^	21.26 ± 1.39^c^
AS + Apagard m-Plus	89.46 ± 2.70	2.08 ± 1.88	2270.39 ± 218.99	347.75 ± 28.65^ad^	15.34 ± 0.58^ad^
AS + Remin Pro	87.80 ± 2.23	1.79 ± 2.03	2250.13 ± 253.76	365.84 ± 37.99^bd^	16.27 ± 0.31^bd^
AS + Clinpro Tooth Crème	88.51 ± 3.74	2.32 ± 3.25	2157.42 ± 274.03	450.38 ± 49.65^c^	20.93 ± 0.96^c^

*LD_d_: ANOVA—no difference between products (p = 0.062, p > 0.05).

^α^LD_d_–LD_r_: ANCOVA—main effects model treatment and LD_d_ as covariate. No difference between products (p > 0.05). Effect of LD_d_ significant (p < 0.0001).

^β^ZD_d_: ANOVA—no difference between products (p > 0.05).

^γ^ZD_d_–ZD_r_: ANCOVA—main effects treatment and ZD_d_ as covariate. Pairwise comparisons with Sidak adjustment. Highly significant differences between products (p < 0.0001). Effect of ZD_d_ significant (p < 0.0001).

^abcd^same superscripts in column denote means that are not significantly different (p > 0.05). Differences between all other means highly significant (p < 0.0001).

^δ^%R: ANCOVA—main effects model untransformed values with treatment and ZD_d_ as covariate. Differences across treatment highly significant (p < 0.0001) and effect of ZD_d_ also significant (p = 0.02).

^abcd^Same superscripts in column denote means that are not significantly different (p > 0.05). Differences between all other means highly significant (p < 0.0001). All differences between treatments were measured using pairwise comparisons with a Sidak adjustment.

No concordance was evident between the SMH and TMR analyses with regard to ranked treatment effect. Accordingly, correlation between %SMHR and %R was not significant, either using treatment means or individual sample values (treatment means: Spearman’s ρ = 0.301, p = 0.456; individual sample values: Spearman’s ρ = 0.246, p = 0.073) (Table 5). Hence, this null hypothesis was also rejected. However, a strong and significant correlation between water-soluble calcium concentration of remineralisation solutions and the corresponding treatment mean %R values was observed (Spearman’s ρ = 0.857, p = 0.007) (Table 5). No significant correlation was found between water-soluble calcium concentration and the corresponding mean %SMHR values (Spearman’s ρ = 0.619, p > 0.05).

**Table 5:** Correlations between %R and %SMHR, and between %R and bioavailable calcium.

	%R (treatment means)	%R (samples)
**%SMHR**		
Spearman's Rho	0.301	0.246
Sig	> 0.05	> 0.05
N	8	56
**Bioavailable calcium**		
Spearman's Rho	0.857	–
Sig	< 0.01	–
N	8	–

## Discussion

As seen in previous studies, the positive correlation between %R and water-soluble calcium demonstrated that remineralisation of enamel subsurface lesions was calcium-limited^7,30–32^. Of the treatment groups, the Tooth Mousse Plus and Tooth Mousse remineralisation solutions were found to produce the highest and second highest %R and contain the highest and second highest water-soluble calcium levels, respectively. These solutions contained casein phosphopeptides (CPP), which are calcium stabilisers and biomimetics of salivary statherin that reversibly form nanocomplexes with calcium and phosphate ions to prevent nucleation of insoluble calcium-containing phases in solution^9,33^. The highly soluble casein phosphopeptide nanocomplexes create a metastable solution supersaturated with respect to the solid calcium phosphate phases, promoting apatite crystal growth in the enamel subsurface lesion^34,35^. While calcium was present in all remineralisation solutions, those with unstabilised calcium (Age Defying, Apagard m-Plus, Remin Pro and Clinpro Tooth Crème) had relatively low amounts detected in a stable water-soluble form. This can be attributed to solubility limits imposed by individual product formulations, or the precipitation of poorly soluble calcium-containing phases in solution^36^. The products containing hydroxyapatite as an active ingredient, Apagard m-Plus and Remin Pro, produced only a slight increase in remineralisation compared to the AS control solution and were not significantly different to the level of remineralisation produced by the standard fluoride toothpaste (Colgate Total).

Enamel subsurface remineralisation (%R) was not significantly correlated with change in surface microhardness (%SMHR). While the present study is the first to test correlation between remineralisation (%R) and %SMHR, a similar discordance between TMR and SMH parameters has been reported when assessing demineralisation^37–40^, and when differentiating treatment effect of various remineralisation therapies^15,22–24^. In the present study, a significant factor influencing %SMHR measurements was the activity of remineralisation treatments on the enamel surface. Although the Age Defying remineralisation solution produced the highest %SMHR, it was observed to have produced extensive surface precipitates (see Figs. 1 and 2). The interpretation of microhardness testing alone may have suggested the superior %SMHR of the Age Defying solution was related to a superior level of remineralisation. Rather, it was the irregular calcium, phosphate, and fluoride-containing surface precipitates that falsely yielded relatively high %SMHR values. As numerous remineralisation technologies contain calcium and phosphate, an appropriate analysis must factor in the possibility of enamel surface precipitation or promotion of calculus formation^8^. In this case, %SMHR failed to accurately represent remineralisation and, thereby, the ‘recovery’ of demineralised enamel was misleading and obfuscated the use of microhardness testing to assess the potential therapeutic benefit of these agents to remineralise enamel subsurface lesions. Furthermore, the treatment that produced the highest enamel subsurface remineralisation (Tooth Mousse Plus), produced only a moderate increase in %SMHR, which was not significantly different to that produced by the positive control. The Tooth Mousse Plus and Tooth Mousse solutions both contained CPP-stabilised amorphous calcium phases which promote mineral gain throughout the lesion body^7,34,41^. As the CPP have a preferential binding affinity for certain faces of the apatite crystals^42^, these interactions prevent complete mineralisation of superficial enamel and help maintain diffusion channels to the subsurface enamel, resulting in relatively less %SMHR and the greatest subsurface remineralisation. The binding of the CPP predominantly to the (100) and (010) faces of the apatite crystals while allowing crystal growth along the c-axis also suggests the peptides promote anisotropic crystal growth to remineralise the enamel crystals as they were originally produced during amelogenesis^42^. Furthermore, the deeper level of remineralisation by the CPP-ACP/F technology can be related to the formation of electroneutral nanocomplexes around 4 nm in size which can diffuse relatively unhindered by enamel surface charge and tortuosity to reach the depth of the lesion^35^. Hence, %SMHR would not comprehensively reflect the full treatment effect of CPP-ACP/F-promoted remineralisation.

Demineralisation and remineralisation of caries lesions are subsurface phenomena. A relatively highly mineralised layer persists in the surface region of carious enamel due to the presence of the acquired enamel pellicle and supersaturation of the superficial lesion fluid, particularly in the presence of fluoride^43^. The thickness of this mineralised surface layer varies in depth up to 130 µm in vivo^44^. Under the microhardness testing conditions in the present study, the Vickers indenter penetrated no more than 7 µm into the enamel surface. In a study by Creeth et al.^26^ investigating remineralisation of caries-like lesions, the Knoop indenter penetrated approximately 4 µm into demineralised enamel. Similarly, an in vitro study that assessed remineralisation of enamel subsurface lesions by calcium- and fluoride-containing toothpastes used a Knoop indenter that penetrated enamel no more than 6 µm^28^. Using standard force, the typical depth of penetration into enamel of Knoop and Vickers indenters is relatively small when experimenting on subsurface lesions of 100 µm depth, and considerably less than the depth of the mineralised surface layer of natural carious lesions. Hence, surface microhardness has limited relevance for measurement of subsurface mineral gain and is more appropriate to measure the mechanical properties of superficial enamel without surface depositions^45^.

In conclusion, enamel subsurface lesions treated by calcium-containing remineralisation technologies in an in vitro remineralisation model, without intermittent demineralisation, were assessed using %SMHR and %R obtained from SMH and TMR measurements, respectively. The %SMHR measurements were not significantly correlated with the %R measurements, while the water-soluble calcium levels in the remineralisation solutions were positively correlated with %R. Surface precipitations resulting from treatments influenced %SMHR measurements and subsurface mineral gain was not accurately represented by %SMHR. The results of the present study demonstrate that %SMHR values should not be used to quantify subsurface remineralisation of caries-like lesions and should be interpreted with caution. TMR remains the more reproducible and appropriate technique to assess remineralisation of caries-like lesions ex vivo due to its relevance, sensitivity and information provided on lesion mineral distribution.

## Materials and methods

### Tooth collection and sample preparation

Human molar teeth were collected from general dental practices after informed patient consent and with approval from the Human Research Ethics Committee of The University of Melbourne (HREC #1033189). We confirm that collection and use of the extracted teeth were in accordance with the relevant guidelines of the Declaration of Helsinki—Ethical principles for medical research involving human subjects. Extracted teeth were sterilized by immersion in 10% (v/v) neutral phosphate buffered formalin for a minimum of two weeks. Teeth included in the study had relatively flat buccal or lingual coronal surfaces free from fluorosis, cracks, irregularities and white spot lesions. Teeth were cleaned of remaining soft tissue, calculus and alveolar bone using a scalpel and the enamel surfaces were polished to a fine mirror finish using a slow speed contra-angle hand piece and 3M Soflex discs (3M, St Paul, USA). The polished enamel surfaces were sectioned from the crowns into blocks approximately 4 × 8 mm using a slow-speed water-cooled Minitom diamond peripheral saw (Struers, Radiometer, USA). The mid-part of the block was ground flat with 1200-, 2400- and 4000-grit silicon carbide lapping paper (Struers, Radiometer, USA) sequentially on a RotoPol-21 grinding/polishing machine with a RotoForce-4 module (Struers, Radiometer, USA) to provide a flat, smooth surface for microhardness testing. Baseline surface microhardness of each enamel block was measured (Vickers diamond, three indentations, 1 N load, 10 s dwell time; MHT-10, Anton Paar, Graz, Austria) to exclude slabs with a Vickers Hardness Number (VHN) outside the normal enamel tissue range of 250–360 VHN^46^. Enamel blocks were then painted with acid-resistant nail varnish (Red 745, Revlon, USA) exposing a window of enamel that was demineralised to produce an artificial subsurface lesion of approximately 1 × 7 mm in area and 100 μm depth according to the protocol described by White^47^ and modified by Reynolds^41^. Each block was sectioned using a water-cooled diamond peripheral saw into two half blocks; one used as the control half-block and the other used as the experimental half-block. Experimental half-blocks had their cut surfaces painted with acid resistant nail varnish and control half-blocks had the nail varnish removed with a scalpel. All half-blocks were then stored in labelled microcentrifuge tubes with a drop of distilled deionised water (DDW).

### Remineralisation protocol

The experimental half-blocks were randomly allocated into 8 groups (A–H, n = 7). Sample size was calculated by estimating an effect size of 5 ± 2% with α = 0.05 to achieve power of 90%. Group A (AS alone) served as the no-product control group, and Group B (AS + Colgate Total) served as the standard fluoride toothpaste positive control group. The remineralisation potential of various commercially available products with calcium-containing remineralisation agents were tested on groups C, D, E, F, G and H (Table 1). The commercial products in Groups B–H were diluted in artificial saliva (AS: 20 mM HEPES buffer, 50 mM NaCl, 0.5 mM CaCl_2_, 0.5 mM Na_2_HPO_4_ adjusted to pH 7.0) as appropriate (see Table 1 for active ingredients). The products containing fluoride were diluted according to the labelled fluoride content to standardise the fluoride concentration at 100 ppm, and products not containing fluoride were diluted 1:10 in AS. Remineralisation solutions for groups B–H were prepared by thoroughly mixing the product with AS using a vortex (250VM; Hwashin Technology Co., Korea) until the product was entirely suspended in AS. Experimental half-blocks were suspended in 2 mL of AS/remineralisation solution in an incubator at 37 °C for 24 h after which the half-blocks were washed thrice with DDW, blotted dry and immersed in fresh AS remineralisation solution for 24 h at 37 °C. This process was repeated for a total period of 10 days. At the end of the treatment period, experimental half-blocks had their nail varnish removed and were stored in labelled, humidified microcentrifuge tubes.

### Calcium analysis of remineralisation solutions

Each remineralisation solution (A-H) was assessed in triplicate for water-soluble calcium content using ion chromatography. Water-soluble ion concentrations were measured from supernatants of fresh remineralisation solutions after centrifugation at 16,000*g* for 10 min at 18 °C. An ion chromatography system (Dionex Corporation, CA, USA) equipped with a cation column measured ion concentrations of collected supernatants following acid extraction (0.01 M HNO_3_) and filtration (0.2 μm, Minisart, Sartorious, Victoria, Australia) as described by Shen et al.^7^.

### Surface microhardness

Following remineralisation, treated half-blocks were matched with their control half-block and assessed for Vickers surface microhardness using a MHT-10 microhardness tester (Anton Paar, Graz, Austria) viewed through a DMLP optical microscope (Leica Microsystems, Wetzlar, Germany) connected to a DFC 320 digital camera (Leica Microsystems, Wetzlar, Germany). The Vickers indenter was applied to the enamel surface at a load of 0.5 N with a dwell time of 10 s. A total of five indentations were made on the flattened surface of each half-block; three across the control or treated half-block lesion, one above the lesion window, and one below the lesion window. The indentations were spaced a minimum distance of 100 μm apart. Images of the indentations were obtained using IM50 Image Manager microscope software (Leica Microsystems, Wetzlar, Germany) and analysed using ImageJ software (National Institutes of Health, version 1.43 for Microsoft Windows). The indentation lengths along the vertical and horizontal axes were recorded, and the Vickers hardness number (VHN) for each enamel surface was calculated using the formula^48^: VHN = 1854.4 × P/d^2^, where P = load (g) and d = mean of diagonals of indentation (μm). The mean VHN for specific enamel surfaces was calculated for each enamel block. %SMHR for each enamel block was determined using the formula^49^: %SMHR = (VHN_r_ − VHN_d_)/(VHN_s_ − VHN_d_) × 100%, where VHN_s_ = mean surface microhardness of sound enamel, VHN_d_ = mean surface microhardness after demineralisation, and VHN_r_ = mean surface microhardness after treatment.

### Scanning electron microscopy

A representative treated half-block from each group was examined under a field-emission scanning electron microscope (FE-SEM) (Quanta 200F, FEI, Oregon, USA) at the Ian Holmes Imaging Centre (Bio21 Institute, Melbourne, Australia). The half-block was dried with compressed air and placed in a fume hood overnight at room temperature. The half-blocks were then mounted and examined under in high vacuum at 10 kV, spot size 2 and pressure of 0.8 mbar. Treated lesions of each experimental half-block and a control half-block were imaged at 5000× magnification.

### Transverse microradiography

Following surface microhardness and SEM analysis, all corresponding experimental and control half-blocks were matched, dehydrated, embedded in cold-curing methyl methacrylate resin (Paladur, Heraeus Kulzer, Germany) and processed for TMR as described previously^50^. Transverse sections of embedded enamel half-blocks were radiographed using Microchrome High Resolution glass plates (3 × 3 × 0.06 in, Microchrome, Tech Inc., CA, USA) and copper Kα radiation at 20 kV, 30 mA for 8 min, alongside an aluminium step wedge with 7 × 37.5 μm thick increments. Microradiographs were developed and imaged as described by Fernando et al.^50^, with the resulting images of radiographed enamel half-blocks assessed for lesion depth (LD_d_ = demineralised control lesion and LD_r_ = remineralised lesion) and densitometric profiles. Volume percent mineral (vol%min) of control lesions, treated lesions and adjacent sound enamel was determined using the equation of Angmar et al.^51^, the linear absorption coefficients of aluminium, organic matter plus water and apatite (131.5, 11.3, and 260.5, respectively), and the section thickness. Through trapezoidal integration, the difference in vol%min between the median sound enamel and remineralised lesion (ΔZ_r_) and median sound enamel and control demineralised lesion (ΔZ_d_) was determined. Percent remineralisation (%R) was then calculated according to the formula^41^: %R = (ΔZ_d_ − ΔZ_r_)/ΔZ_d_ × 100%.

### Data analysis

All statistical analyses were performed using statistical analysis software (Minitab Inc. version 16, State College, PA, USA and IBM SPSS Statistics for Windows, Version 26.0 SPSS IBM Corp. Armonk, NY, USA). For each parameter (VHN_s,d,r_, VHN_r_–VHN_d_, %SMHR, LD_d,_ LD_d_–LD_r_, ΔZ_d_, ΔZ_d_–ΔZ_r_, %R) normality of residuals was tested using normal probability plots and the Shapiro–Wilk test, and homogeneity of variance of residuals was tested using Levene’s test. For surface microhardness parameters, differences between means for VHN_s_ were measured using the Kruskal–Wallis test and differences in means for both for VHN_d_ and VHN_r_ were measured using ANOVA. Differences in mean %SMHR values were measured using ANCOVA with VHN_s_-VHN_d_ as a covariate. For lesion parameters measured with TMR, differences in both means for LDd and ZDd were measured using ANOVA. Differences in mean LDd–LDr values were measured using ANCOVA with LDd as the covariate. Differences in means for both ZDd-ZDr and %R were measured using ANCOVA with ZDd as the covariate. All differences between each pair of treatments were measured using pairwise comparisons using a Sidak adjustment. Correlations between mean %R and both mean %SMHR and individual values for %SMHR, between %R and water-soluble calcium concentrations in the remineralisation solutions, as well as between %SMHR and water-soluble calcium concentrations were measured using Spearman’s rank correlation coefficient. The significance level for all statistical tests was set at α = 0.05.
